# Improved optical properties of perovskite solar cells by introducing Ag nanopartices and ITO AR layers

**DOI:** 10.1038/s41598-021-93914-1

**Published:** 2021-07-15

**Authors:** Yangxi Chen, Chaoling Du, Lu Sun, Tianyi Fu, Ruxin Zhang, Wangxu Rong, Shuiyan Cao, Xiang Li, Honglie Shen, Daning Shi

**Affiliations:** 1 College of Science, Nanjing University of Aeronautics and Astronautics, Nanjing, 211100 People’s Republic of China; 2 Key Laboratory of Aerospace Information Materials and Physics, Ministry of Industry and Information Technology, Nanjing, 210016 People’s Republic of China; 3 College of Materials Science and Technology, Nanjing University of Aeronautics and Astronautics, Nanjing, 211100 People’s Republic of China

**Keywords:** Nanoscience and technology, Optics and photonics

## Abstract

Embedded noble metal nanostructures and surface anti-reflection (AR) layers affect the optical properties of methylammonium lead iodide (CH_3_NH_3_PbI_3_) perovskite solar cells significantly. Herein, by employing a combined finite element method and genetic algorithm approach, we report five different types of CH_3_NH_3_PbI_3_ perovskite solar cells by introducing embedded Ag nanoparticles within the CH_3_NH_3_PbI_3_ layer and/or top ITO cylinder grating as an AR layer. The maximum photocurrent was optimized to reach 23.56 mA/cm^2^, which was 1.09/1.17 times higher than Tran’s report/ flat cases. It is also comparable with values (23.6 mA/cm^2^) reported in the literature. The calculations of the electric field and charge carrier generation rate of the optimized solar cell further confirms this improvement than flat cases. It attributes to the synergistic effect of the embedded Ag nanoparticles and ITO AR layer. The results obtained herein hold great promise for future boosting the optical efficiency of perovskite solar cells.

## Introduction

Solar cells have been extensively designed and optimized to improve the efficiency of solar energy harvesting. Among various solar cells, perovskite-based solar cells have impressive power conversion efficiency, which has improved from 3.8% to exceeding 25% in recent years^1,2^. Due to the unique optical and electrical properties of perovskites, perovskite-based solar cells are considered as one of the best candidates to replace traditional Si or GaAs solar cells. The bandgap (*E*_*g*_) of methylammonium lead iodide (CH_3_NH_3_PbI_3_) is approximately 1.5 eV, which enables efficient absorption in the visible light region^3,4^. However, the intrinsic low absorption of CH_3_NH_3_PbI_3_ surpassing 770 nm will decrease the performance of perovskite solar cells^5^. Recent progress has shown that the light absorption of perovskite solar cells can be increased by introducing plasmonic metal nanostructures, eventually enhancing the photocurren t (*J*_*sc*_)^6–10^. The excitation of localized surface plasmon resonance (LSPR) of plasmonic nanostructures further improves the light absorption of perovskites, and both radiative and non-radiative effects of LSPR can enhance the optical properties of solar devices^11,12^. Additionally, sub-wavelength dielectric nanostructures have received considerable attention as anti-reflection (AR) layers^13–17^. However, to the best of our knowledge, only few studies have been reported on the enhancement of the optical properties of perovskite solar cells by the introduction of both AR grating and plasmonic metal nanostructures. Thus, research on the improvement of the light absorption, *J*_*sc*_, and charge carrier generation rate of perovskite solar cells is crucial. Compared with experiments, numerical simulation provides a faster and easier method for this investigation and has been successfully applied to optimize the optical properties of perovskite solar cells. Tran et al. employed a finite-difference-time-domain (FDTD) method and achieved a maximum *J*_*sc*_ of ~ 21.5 mA/cm^2^ by embedding Ag nanocubes into the perovskite layer at normal incidence^18^. Moreover, using the same simulation method, Heidarzadeh et al. predicted a maximum *J*_*sc*_ of ~ 22.5 mA/cm^2^ utilizing Au dimers^19^. However, it is still difficult to achieve accurate optimal results from the optimized geometry parameters predicted based on physical intuition and experience only. Recently, by optimizing the *J*_*sc*_ of solar cells, genetic algorithm (GA) has been proven as an efficient method along with FDTD/finite element method (FEM) to optimize the geometric parameters of light-trapping nanostructures^20,21^.

To further improve the optical properties of flat perovskite thin-film solar cells, herein, we proposed five different types of perovskite solar cells (I–V) by introducing embedded Ag nanoparticles and/or top ITO AR grating into perovskite solar cells. Geometries and periods of the structures were optimized by the co-simulation of FEM and GA to achieve optimized optical properties, such as *J*_*sc*_, and performance of solar cells.Furthermore, wavelength-dependent electric field and charge carrier generation rate were calculated to understand the light propagation behavior in these cells. Moreover, a comparison between the *J*_*sc*_ and absorption enhancement of the optimized solar cell and a flat perovskite thin-film solar cells presented. Finally, we have also discussed the effect of incident angle on the *J*_*sc*_ and absorbed energy of the proposed cells.

## Simulation structures and methods

**Figure 1:**
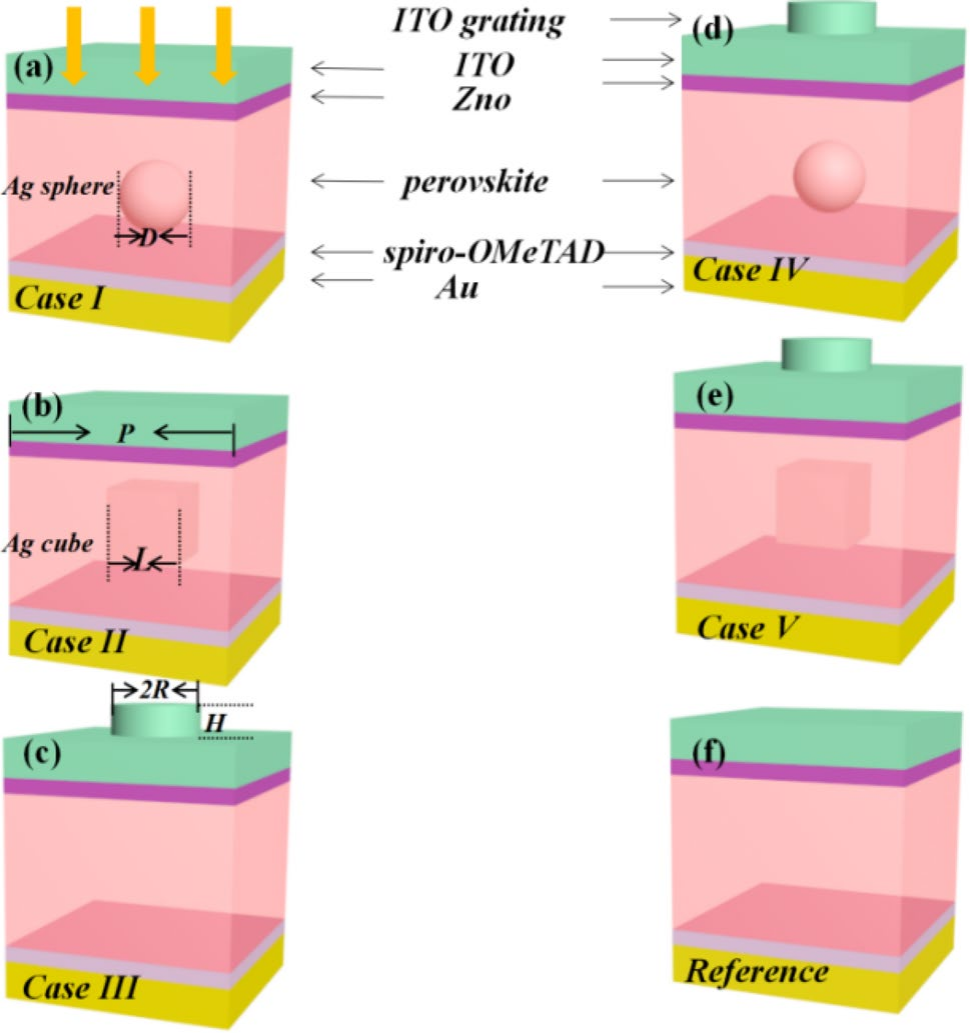
3D schematic of the unit cell geometries of different types of perovskite solar cells proposed herein.

Optical properties of the proposed perovskite solar cells were simulated using a commercially available FEM package [COMSOL Multiphysics 4.1 (http://www.comsol.com) with the RF module]. The simulated 3D model of each the concerned five types of perovskite solar cells are schematically shown as Fig. 1 along with that of flat reference cell. Every cell consists of multiple layers, that is, from top to bottom, 100 nm ITO as a transparent front electrode, 30 nm ZnO as an electron transport layer (ETL), a 300 nm perovskite layer as an absorbing layer, 50 nm spiro-OMeTAD as a hole transport layer (HTL), and an 80 nm Au layer as a back contact. Thanks to the developing of sputtering, CVD/PVD, or ALD deposition techniques in experiments^15,17,22,23^, thin ETL or HTL have been adopted herein. In cases I and II, Ag nanospheres and nanocubes were embedded in the middle of the perovskite layer, respectively. In case III, an integrated ITO cylinder grating was introduced as an AR layer on the top surface of the ITO electrode. Based on this configuration, embedded Ag nanoparticles were incorporated in cases IV and V to further improve the optical properties of the flat perovskite solar cells.Moreover, a flat perovskite solar cell(without the ITO grating and Ag nanoparticles) was used as a reference. Herein, because the proposed devices are substantially thin (only ~ 560 nm thick excluding the top cylinder grating), the electron–hole recombination rate is negligible. During the simulation, the wavelength-dependent optical constants of CH_3_NH_3_PbI_3_ and spiro-OMeTAD were obtained from the data reported in the literature^24,25^, whereas those of the remaining materials were directly extracted from the COMSOL optical material database. The refractive index of surrounding medium was set as air for all the concerned cells. To perform simulations for each type of proposed solar cells, incident light was irradiated along the *z* direction from the top cell surface, which polarized along the *y*-direction. To avoid spurious reflections, an artificial domain of a 400 nm perfect match layer was applied along the *z*-direction,and Floquet periodicity conditions were employed along both the *x*- and *y*-axes. The maximum mesh element size was set to 1/7 of the maximum incident light wavelength for all the simulated domains. Then, the electromagnetic field and light absorption of one unit cell were calculated using the well-known Helmholtz equation: $\nabla  \times \nabla  \times E - k_{0} ^{2} \varepsilon _{{\text{r}}} E = 0$^26^. Here, $k_{0}$ is the wave vector of the incident light, and $\varepsilon _{{\text{r}}}$ is the dielectric permittivity of the related medium. Normalized power absorption ($P_{{abs}}$) of each cell was determined by $P_{{abs}}  = \frac{1}{2}\omega \left| {E(\omega )} \right|^{2} \text{Im} \varepsilon ((\omega ))$, where |*E*(*ω*)| and $\text{Im} \varepsilon (\omega )$ are the normed |*E*| at an angular frequency $\omega$ and the imaginary part of the dielectric constant of the corresponding material, respectively. The corresponding photocurrent was evaluated using $J_{{{\text{sc}}}} (\lambda ) = \int\limits_{{300}}^{{800}} {\frac{{e\lambda }}{{hc}}P_{{abs}} (\lambda )I(\lambda )d\lambda }$^17,27^, where *e*, *h*, *c*, and $I(\lambda )$ are the charge of an electron, Planck’s constant, the speed of light in free space, and AM(1.5) solar cell spectrum, respectively. During simulations, we used the AM(1.5) solar spectrum for incident light. The corresponding charge carrier generation rate at each wavelength was calculated by $G = \frac{{P_{{abs}} }}{{\hbar \omega }} = \frac{{\left| {E(\omega )} \right|^{2} \text{Im} \varepsilon ((\omega ))}}{{2\hbar }}$^28^.

### GA method

To optimize the optical properties of solar cells, GA is an effective method that can be combined with FEM or FDTD simulation in addition to topology and neural network optimization^20,21,29,30^. This is attributed to the strong global searching ability and independence of the initial structure of GA^20,21^, which starts with a randomly generated initial population. Subsequently, GA operates on a population of artificial chromosomes, which represents a solution to the related problem. Bit-string chromosomes consist of a string of genes that participate in GA. The fitness value of every solution can be evaluated by the fitness function. A higher fitness value of the solution indicates more adaptability of the solution to the natural environment and a larger probability to produce offspring. The selected variables of the solution based on the fitness value are decoded to the binary code in GA. Subsequently, the binary variables randomly mate and produce new offspring followed by crossover and mutation. The average fitness value of the offspring has a larger probability, which is higher than that of the older generation. During evolution, the entire population evolves toward an optimal solution.

### Co-simulation of FEM and GA

**Figure 2:**
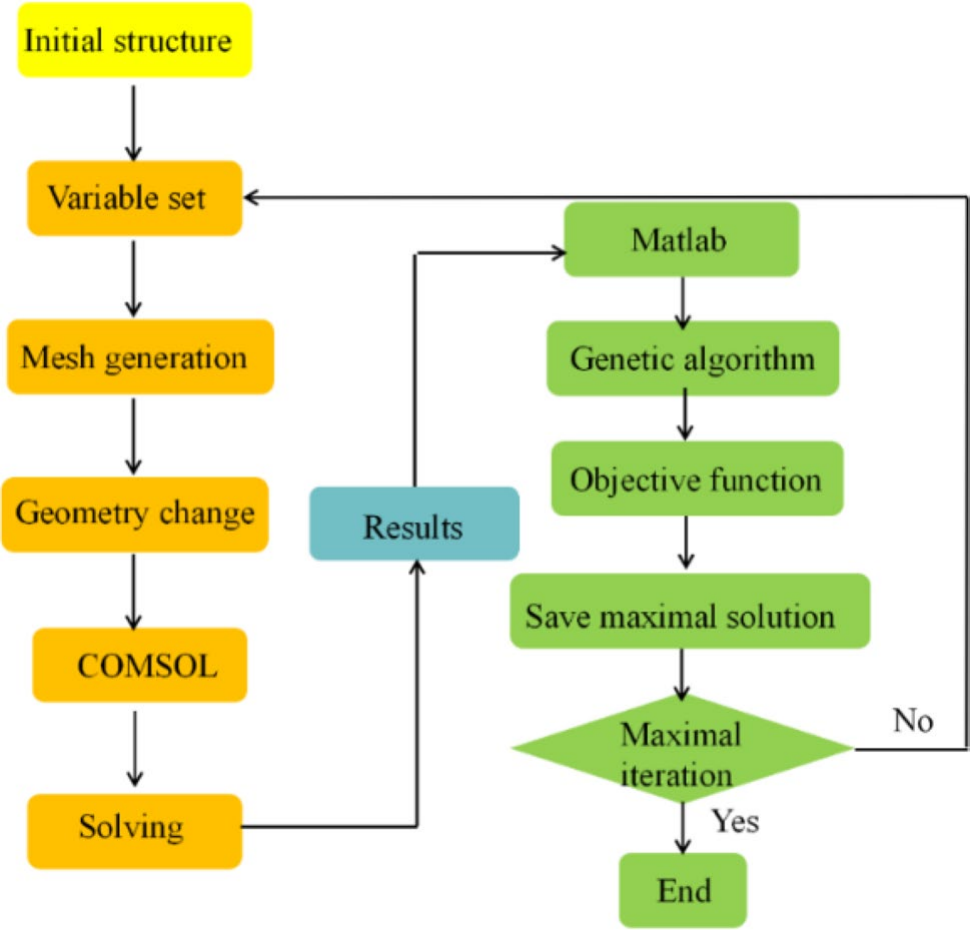
Flow chart of the co-simulation of COMSOL and GA used in this study.

The flow chart of the co-simulation of FEM and GA is presented in Fig. 2. The random initial structure constructed using COMSOL as the optimized process is independent of the initial structure. Simulated data of the initial structure are taken as input parameters for GA optimization. Then, GA generates new geometric parameters, which are employed as the new input parameters in COMSOL to perform the simulation. Subsequently, optimization recycling starts until the iteration times of evolution are reached.

In this study, Ag was chosen as the plasmonic nanoparticle material to enhance the optical properties of the proposed solar cells, essentially because Ag nanoparticles have a larger scattering cross-section than that of Au nanoparticle sat the same geometric parameters^31^. The reason for using an ITO nanocylinder grating as the AR layer is that nanocylinder-shaped ITO has been successfully fabricated by UV-nanoimprint lithography and is widely used in solar cells to enhance incident light absorption^14,32^. In the first step of simulation, we optimized the geometric parameters, including sphere diameter (*D*), cube length (*L*), and corresponding period (*P*),of the embedded Ag nanospheres and nanocubes for cases I and II. *P* was set in the range from 150 to 1000 nm according to the literature, which showed that optimal *P* was several hundreds of nanometers^18^. Furthermore, *D* and *L* were set to be smaller than the corresponding *P*.Using the co-simulation of FEM and GA, optimal *P*, *D* and *L* were determined by optimizing *J*_*sc*_ at different *P*, *D*, and *L* for cases I and II. To explore the optical effect of the AR layer, case III was introduced. Its optimal *P*, ITO grating height (*H*), and radius (*R*) were then assessed by the co-simulations of FEM and GA. For the integrated cases IV and V, multiple variables, namely, *D, L*, *H*, *R*, and their *P* (*P* of the ITO nanocylinder and Ag nanoparticles was supposed to be equal for simplicity), were simultaneously optimized. During the optimization of each cell, the number population and iteration evolution were set at 30 and 25, respectively.

## Results and discussion

**Figure 3:**
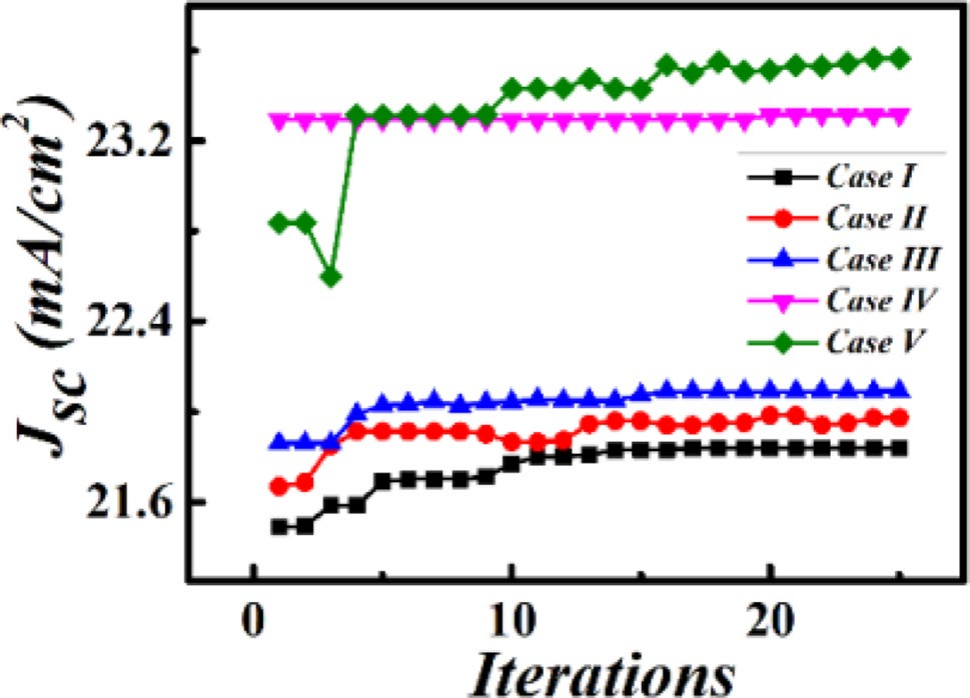
Simulated *J*_*sc*_ versus simulation iteration times for the five proposed cases shown in Fig. 1.

Simulated *J*_*sc*_ of all the proposed cells is plotted in Fig. 3. As shown in Fig. 3, the maximum *J*_*sc*_ for cases I and II reaches 21.84 and 21.97 mA/cm^2^ with the *D* and *L* of 288.71 and 110.69 nm and *P* of 542.33 and 526.19 nm, respectively. The slightly larger maximum *J*_*sc*_ for case II is attributed to the sharper corners of the cube, further leading to a stronger electromagnetic field than that of the sphere in case II^18^. In case III, upon introducing the AR layer, the optimal *J*_*sc*_ reaches ~ 22.09 mA/cm^2^, which is larger than those for cases I and II,owing to the low absorption and AR effect of dielectric ITO grating. The corresponding optimized *P*, *H*, and *R* are 499.94, 146.89, and 190.22 nm. Moreover, Fig. 3 demonstrates that the simulated *J*_*sc*_ for cases IV and V is further improved, validating the positive role of the integrated configurations with the combination of the AR layer and Ag nanoparticles. The maximum *J*_*sc*_ for cases IV and V is simulated to be 23.31and 23.56 mA/cm^2^ at the optimized *H* of 124.56 and 164.72 nm, *R* of 190.03 and 169.09 nm, *D* and *L* of 268.19 and 120.67 nm, and *P* of 458.04 and 509.45 nm, respectively. Comparative analysis of these results indicates that cases IV and V achieve better light-capturing ability than that of the cells containing only embedded Ag nanoparticles within the perovskite layer or ITO grating on the top surface of the ITO electrode. Hence, the combination of the embedded Ag nanoparticles and the top AR layer is responsible for the enhanced *J*_*sc*_. The *J*_*sc*_ of the flat perovskite solar cell reference (Fig. 1f) with neither the embedded Ag nanoparticles nor the ITO AR grating is calculated to be ~ 20.02 mA/cm^2^, which is 1.17-fold smaller than the maximum *J*_*sc*_ obtained for the optimized case among the proposed five types of cases.

**Figure 4:**
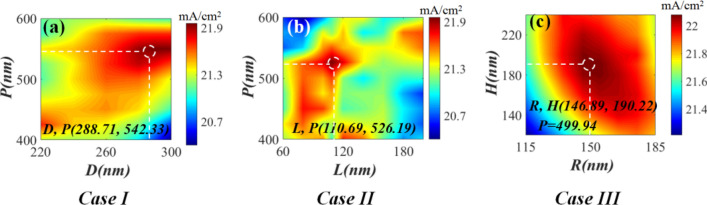
FEM-calculated 2D *Jsc* maps for the cases I (**a**), II (**b**), and III (**c**) shown in Fig. 1.

**Figure 5:**
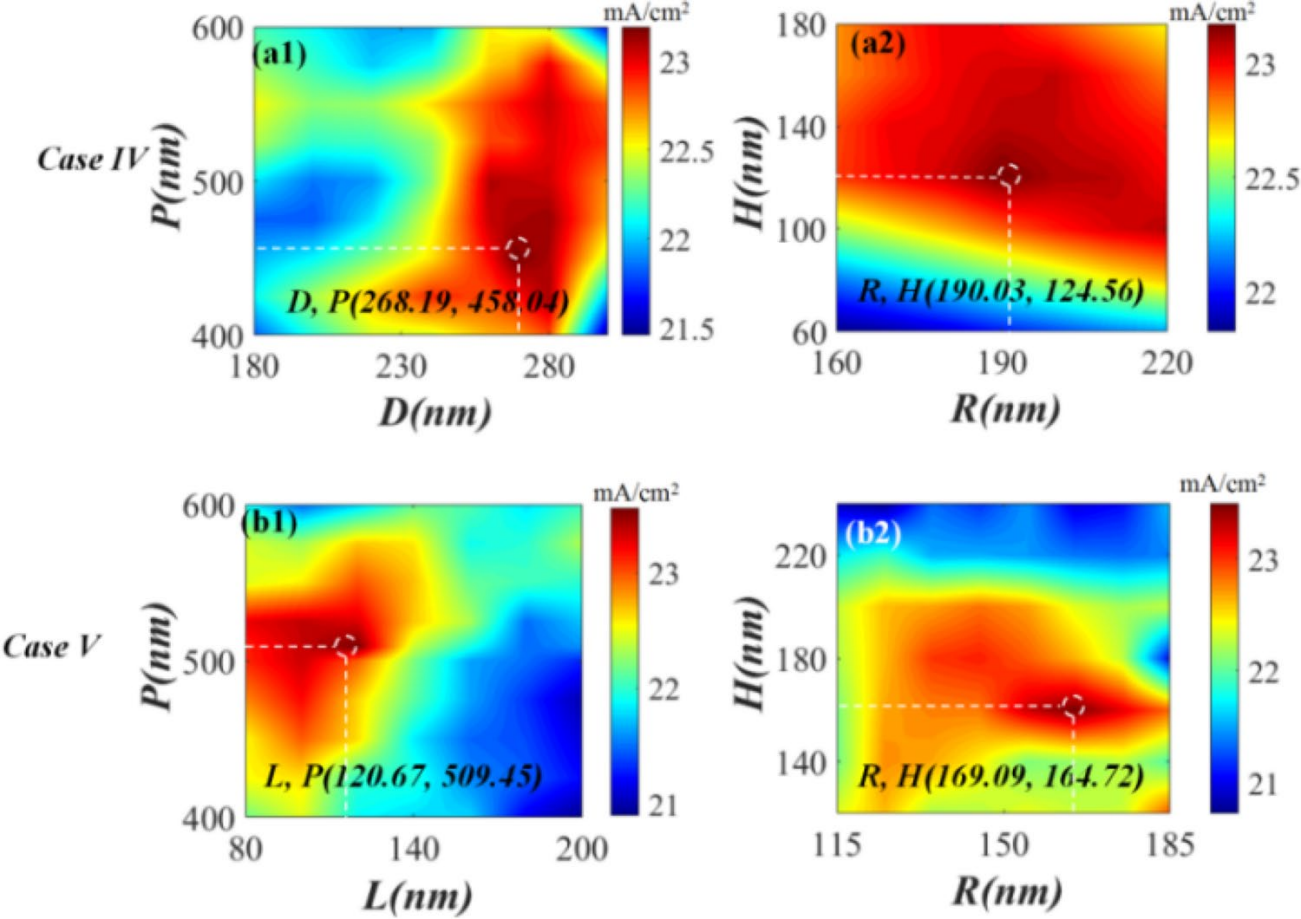
FEM-calculated 2D *Jsc* maps for cases IV (**a**) and V (**b**) presented in Fig. 1.

Subsequently, based on the abovementioned co-simulation of FEM and GA, we performed COMSOL simulation using the obtained optimal geometric parameters to further investigate the variation in *J*_*sc*_ versus the solar cell structure. The acquired 2D map shown in Fig. 4 reveals that the *J*_*sc*_ of cases I, II, and III is sensitive to the sizes of both the embedded Ag nanoparticles in the perovskite active layer (Fig. 4a,b) and the top AR layer (Fig. 4c). This demonstrates that neither the larger size of the Ag nanoparticles nor the larger size of the AR layer is responsible for obtaining a larger *J*_*sc*_. The maximum *J*_*sc*_ achieved for cases I, II, and III is 21.97, 21.83, and 22.09 mA/cm^2^, respectively, which agrees well with the abovementioned co-simulation results (Fig. 3); the corresponding optimized geometric parameters are presented in Fig. 4a,c. For cases IV and V, the variation in *J*_*sc*_, with respect to the sizes of the top AR layer and embedded Ag nanoparticles, is plotted in Fig. 5. Figure 5a1,b1 show that the obtained *J*_*sc*_ is sensitive to the sizes of the embedded Ag nanospheres (Fig. 5a1) and nanocubes (Fig. 5b1) at the optimized size of the top AR layer. Moreover, Fig. 5a2,b2 demonstrate that at the optimized size and *P* of the embedded nanoparticles, the obtained *J*_*sc*_ varies with the size of the top AR layer. This implies that to achieve a larger *J*_*sc*_, it is not feasible to simultaneously embed larger Ag nanoparticles and introduce a larger AR layer. The maximum *J*_*sc*_ for cases IV and V reaches ~ 23.31 and 23.56 mA/cm^2^, respectively, which agrees well with the results shown in Fig. 3, the corresponding optimized geometric parameters are provided in Fig. 5a,b for guidance. The optimal *J*_*sc*_ for cases I–V is 1.09-, 1.09-, 1.10-, 1.16-, and 1.17-fold higher than that of the reference. The ~ 10% improvement in the optimal *J*_*sc*_ for cases I, II, and III also indicates the contribution of the improved scattering from Ag nanoparticles and enhanced transmittance from the AR layer to absorption; therefore, the *J*_*sc*_ of the reference is comparable to those of these cases. Moreover, the results obtained for cases VI and V reveal that the synergistic effect of both the AR layer and Ag nanoparticles increased the *J*_*sc*_ by ~ 6–7% as compared to those of cases I, II, and III.The above mentioned results further confirm the advantages of the optimized case V.

**Figure 6:**
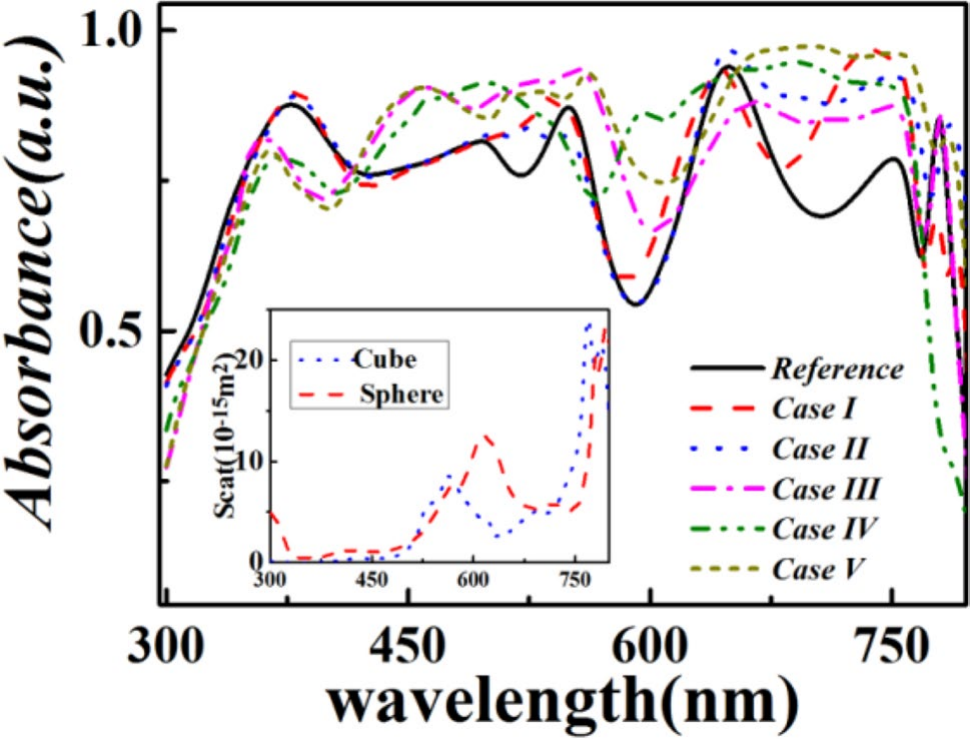
Comparative plots of the optimized absorption spectra of the five proposed cases of solar cells presented in Fig. 1 along with the spectrum of the reference.

Figure S1 presents the absorption spectra of the embedded Ag nanocube in the optimized solar cell of Case V. Its comparison with that of perovskite material of the same cell implies that the parasitic Ag absorption is too weak to be ignored. Then, the absorption spectra of the optimized cells for each case shown in Fig. 1 along with the reference are presented in Fig. 6. For cases I and II, no noticeable absorption enhancement can be observed at wavelengths smaller than 600 nm. However, at larger wavelengths ranging from ~ 600 to 750 nm, significant absorption enhancement occurs for these two cases as compared to that of the reference. Hence, the introduced Ag nanoparticles in cases I and II can compensate for the low intrinsic light absorption of the reference at ~ 600–750 nm. The inset of Fig. 6 illustrates the scattering spectra of the Ag nanospheres and nanocubes obtained from cases I and II, respectively. The spectra show two LSPR peaks at ~ 560(613) and 768(795)nm for the investigated nanospheres (nanocubes), respectively. This further verifies the enhanced absorption for cases I and II at 600–750 nm. In addition, as shown in Fig. 6, absorption enhancement for case III mainly occurs at wavelengths ranging from ~ 415 to 618 nm as compared to those of cases I and II and the reference. This may be owing to the fact that the AR layer reduces the difference between the refractive indices of air and the ITO electrode. This tentatively leads to the transmission of more incident light to the solar cells. For the cases IV and V, it is noticed that enhanced absorption occurs at the incident light wavelengths ranging from ~ 415 to 750 nm as compared to that of the reference. This can be reasonably attributed to the synergistic effect of the grating effect and the LSPR enhancement, which further contributes to the larger incident light-trapping ability of the corresponding cases; hence, the above mentioned *J*_*sc*_ is larger than that of the reference and cases I–III (Figs. 3 and 5). To validate the accuracy of our simulation results, we also compared the absorption spectra of optimized case V between the finer mesh and normal mesh. For finer mesh case, the maximal/minimum mesh element is set to be 10/2.6 nm while the maximum mesh growing rate and curvature rate are set as 1.35 and 0.3, respectively, for the subdomain of Ag nanoparticle. The maximum mesh element of other subdomains is set to vary from 15 to 35 nm to keep convergence. The corresponding number of degrees of freedom reaches 244716. Comparable results are obtained as shown in Fig. S2, which confirms the accuracy of the simulations from another point of view.

**Figure 7:**
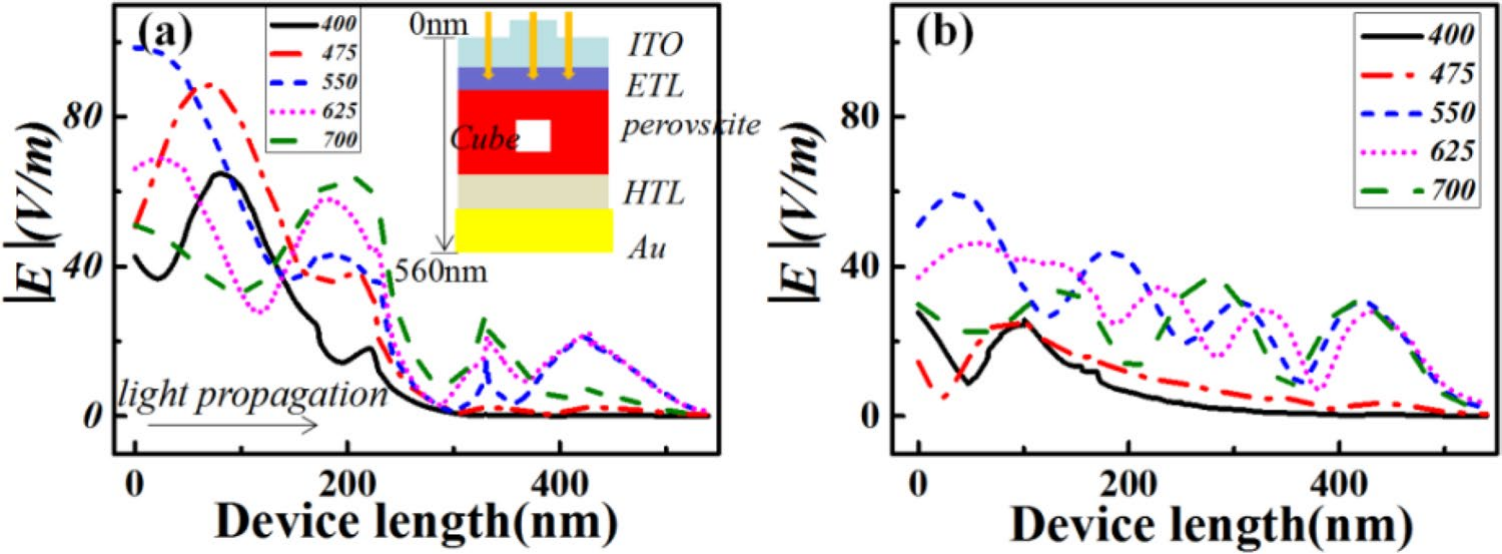
Device position (along with the *z*-direction)-dependent|*E*|profiles of the optimized case V (**a**) and reference (**b**) at several typical incident wavelengths. The upper-right inset of a schematically illustrates the device layer structure.

Normally, the optical absorption of a material directly originates from the corresponding electromagnetic field distributions. The |*E*| profiles of the optimized case V with the largest optimal *J*_*sc*_ among those of the proposed cases were then calculated by solving the Helmholtz equation using COMSOL. Figure 7 shows the calculated |*E*| profiles with respect to the device length, that is, the propagation direction of the incident light (*z*-direction) at five typical incident wavelengths along with that of the reference. For ease of reference, the corresponding distribution maps of both electric fields (Fig. S3a1,b1) and power densities (Fig. S3a2,b2) at incident wavelength 400 and 700nm are presented as Fig. S3 to further reveal how the Ag nanoparticles and ITO nanograting leading to the improved absorption. It can be seen from Fig. 7 that at each of these typical wavelengths, |*E*| in the solar cell increases as compared to that of the reference, which consequently contributes to the larger absorption of case V. For example, at 400nm, the maximum |*E*| of case V was calculated to be 65.02V/m in the middle of the ITO electrode, which matches with Fig. S3 and was ~2.32-fold higher than that of the reference. This enhancement was attributed to the AR layer, which scattered more light into the cell. However, |*E*| drastically decreased with an increase in device length;this indicated low penetration ability of incident light at 400nm. With an increase in the wavelength of the incident light, light does not focus on the top surface of the cell, and then, more light energy is transmitted to the bottom section of the cell. Additionally, at a longer wavelength, such as 700nm, a fringe-shaped profile of the |*E*| along with the device length is observed, as shown in Fig. 7b. This is due to the nearly zero extinction coefficient of perovskite. Thus, the reference has a similar character to that of dielectric materials at longer wavelengths. Moreover, the incident light propagates along the entire device, which interferes with the light reflected by the bottom Au electrode and then forms the observed fringes within the device. However, these fringes are disturbed by the introduction of Ag nanoparticles, and the maximum |*E*| for the optimized case V is significantly enhanced, as shown in Fig. 7a and Fig. S3. This further contributes to the larger absorption of case V than that of the reference (refer to Fig. 6). Comparative analysis of the results presented in Fig. 7a,b confirms that the |*E*| enhancement is because of the LSPR effect of Ag nanoparticles and the AR grating effect, which further validates the absorption enhancement depicted in Fig. 6.

**Figure 8:**
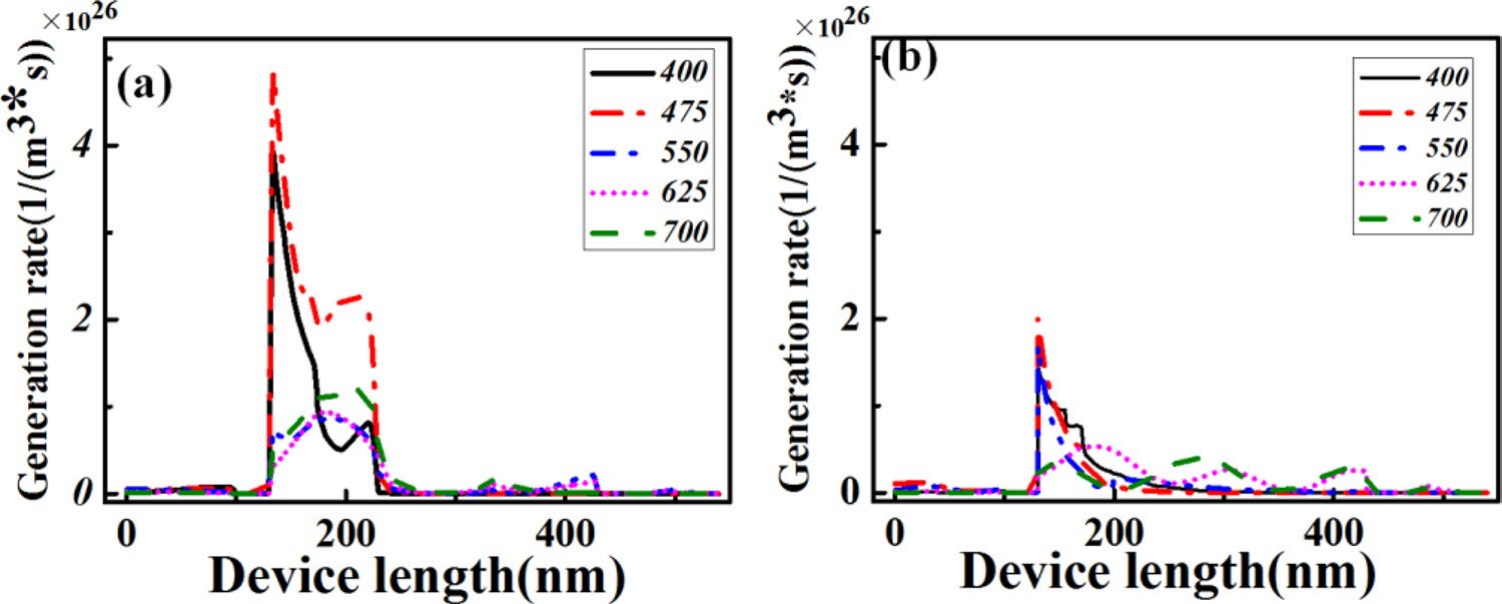
Device position (along the *z*-direction)-dependent generation rate profiles of charge carrier for the optimized case V (**a**) and reference (**b**) at several typical incident wavelengths.

To further estimate the optical performance of the optimized case V, we also calculated the optical generation rate of charge carriers. The obtained wavelength-dependent behaviors of the optimized case V and the reference are plotted in Fig. 8a,b, respectively. The maximum charge carrier generation rate of both the reference and the optimized case V is observed on the top surface of the perovskite material at the incident light wavelength of 475 nm. This is because at 475 nm, the incident light energy (~ 1.6 W/m^2^) is larger than that at other incident wavelengths, according to the AM(1.5) sun spectrum. Furthermore, the related ITO electrode and electron/hole transport layers do not significantly contribute to the generation of charge carriers. The reason for this is the small imaginary part of the optical refractive index of these materials. The maximum charge carrier generation rate for the optimized case V was evaluated to be 4.79 × 10^26^ m^−3^ s^−1^, which is ~ 2.4-fold higher than that of the reference. It is 10^26^ orders of magnitude, which is similar to that previously reported for perovskite and/or Si solar cells^33,34^. Furthermore, the corresponding bandwidth of the carrier generation rate curve is substantially broader at 475 nm. As illustrated in Fig. 8, the charge carrier generation rate is mainly distributed in the entire active perovskite layer at longer wavelengths among the related typical wavelengths. For example, at 700 nm, the maximum carrier generation rate is ~ 1.52 × 10^26^ m^−3^ s^−1^, which is achieved in the middle of the active layer. Additionally, the top surface in case V provides a contribution of 7.13 × 10^25^ m^−3^ s^−1^, which is 2.37-fold higher than that of the reference. This again confirms the advantage of case V as compared to that of the reference.

**Figure 9:**
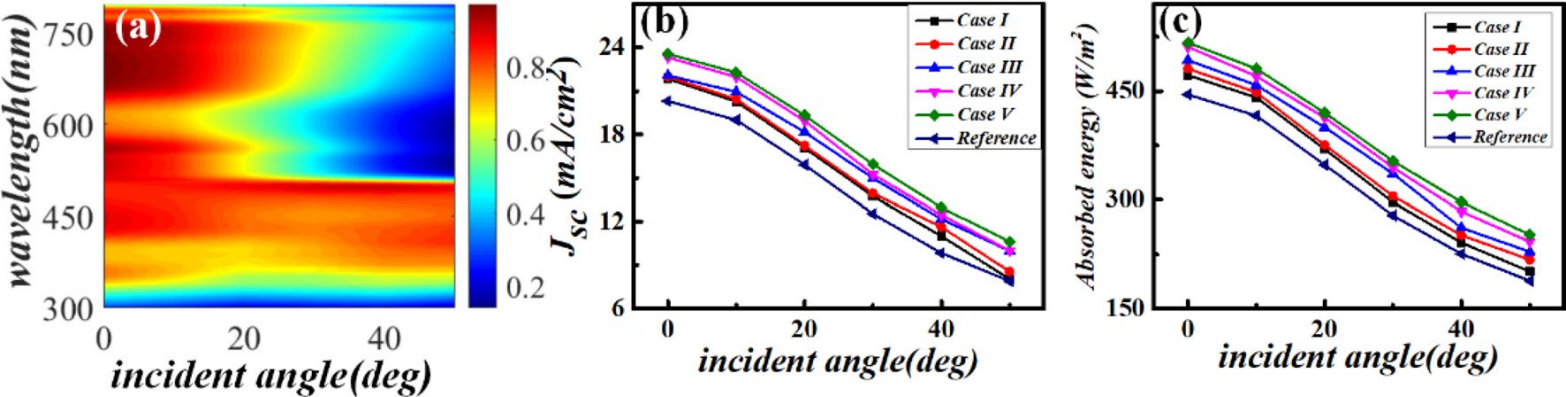
Incident angle-dependent optical properties of the proposed solar cells. (**a**) 2D map of the calculated incident angle-dependent absorption efficiency of the optimized case V. (**b** and **c**) Comparison between the incident angle-dependent optimized *J*_*sc*_ (**b**) and absorbed energy (**c**) of the five proposed solar cells and reference.

The effect of the incident angle on the *J*_*sc*_ and absorbed energy of the proposed cases is presented in Fig. 9. Results (Fig. 9a) show that when the incident angle is varied from 0° to 50°, the absorption efficiency for the optimized case V remains nearly unchanged at wavelengths ranging from 300 to 500 nm. Comparatively, at longer wavelengths, such as in the range from 500 to 800 nm, the absorption efficiency becomes sensitive to wavelength. However, at longer wavelengths (~ 500–800 nm) such as 600 nm, the obtained |*E*| becomes sensitive to the incident angle and its maximum drops from 35 V/m at 0° to 14 V/m at 50°, thus decreasing the corresponding absorption efficiency. These wavelength-dependent behaviors further result in an angle-dependent decrease in the corresponding *J*_*sc*_ and absorbed energy, as shown in Fig. 9b,c, respectively. Figure 9b reveals that the optimized case V exhibits a larger *J*_*sc*_ than those of the other cases at each considered incident angle. The *J*_*sc*_ of each cell decreases with an increase in the incident angle. The maximum *J*_*sc*_ of ~ 23.56 mA/cm^2^ was acquired for the optimized case V at normal incidence. Figure 9c demonstrates that the angle-dependent absorbed energy for the perovskite active layer of each cell is similar to the corresponding angle-dependent *J*_*sc*_. The maximum absorbed energy reaches ~ 516.70 W/m^2^ for the optimized case V at normal incidence. However, it decreases to 251.87 W/m^2^ at 50°. Thus, the maximum *J*_*sc*_ obtained herein is ~ 1.09 fold higher than that achieved by Tran et al. (21.5 m A/cm^2^) at normal incidence^18^. In literature, an optimal *J*_*sc*_ ~ 23.6 mA/cm^2^ was also reported recently by simultaneously using high compact TiO_2_ nanoparticles and front textured AR layer^35^, which is comparable to the present result. Additionally, to avoid the reaction of silver with iodine to rapidly form an AgI like insulating complex, hence severely limiting device performance, we then adopted an TiO_2_ dielectric shell to encapsulate the embedded NSP to work as insulator isolating the NSP from the perovskite layer^36–38^. During the simulations, the adaptive mesh is chosen which is set to vary from 2 to 12 nm for all concerned TiO_2_ nanoshells while the mesh element size of Ag cube is set as 10 nm. The absorption spectra of the optimized solar cell of Case V with which Ag nanocube is encapsulated with different thicknesses *t* of TiO_2_ was plotted as Fig. S4. The corresponding *t* dependent *J*_*sc*_ was shown as the inset of Fig. S4. The final *J*_*sc*_ are revealed to be 23.56 m A/cm^2^, 23.47 m A/cm^2^, 23.13 mA/cm^2^, 23.24 mA/cm^2^, and 23.25 mA/cm^2^ for *t* = 0, 3, 6, 9, and 12 nm, respectively. It demonstrates that TiO_2_ provides a good protection layer to the absorber which doesn’t diminish *J*_*sc*_, further the optical efficiency of the proposed optimized cell of case V. Additional, to better understand the plasmonic effect of TiO_2_ nanoshell, the corresponding distribution maps of both electric fields and power densities of the optimized Case V coated with 12 nm TiO_2_ nanoshell are simulated. The typical results 400 nm and 700 nm are presented at Fig. S5. Unlike the plasmonic effect exponentially stronger as it approaching metal-dielectric-metal sandwich structure elsewhere^39^, the obtained mappings of Fig. S5 doesn’t exhibit resonant oscillation at the semiconductor-dielectric-metal layer. Hence the TiO_2_ nanoshell only show limited impact on the corresponding absorption spectra as illustrated by Fig. S4.

## Conclusion

In this study, the numerical co-simulation of GA and FEM was employed to optimize the optical properties of five different types of perovskite thin-film solar cells. The optimized *J*_*sc*_ for cases I, II, III, IV, and V increased by 1.09-, 1.09-, 1.10-, 1.16-, and 1.17-fold, respectively, as compared to that of the reference. Themaximum *J*_*sc*_ reached23.56 mA/cm^2^, which was achieved for case V containing both the top AR layer (*R:*169.09 nm and *H*: 164.72 nm) and embedded Ag nanocubes (*L*:116.67 nm) within the perovskite layer. The corresponding absorbed energy was calculated to be 516.70 W/m^2^, which was 1.16 times higher than that of the reference. It is concluded that the AR layer improves incident light absorption at shorter wavelengths (~ 415–618 nm) and the embedded Ag nanostructures improve light absorption at longer wavelengths (~ 600–750 nm).The maximum charge carrier generation rate of the optimized solar cell reached up to 4.79 × 10^26^ m^−3^ s^−1^ at the top surface of the perovskite, which is ~ 2.4-fold higher than that of the reference at 475 nm. In addition, a larger *J*_*sc*_ can be achieved at a smaller incident angle when appropriate embedded Ag nanoparticles and top ITO cylinder grating are selected.Considering the vast demand for sustainable and green energy, we believe that the present study holds great promise for the future design and optimization of high-efficiency perovskite-based solar cells.

## Supplementary Information

Supplementary Information.
