# Supplementary material for: Improved optical properties of perovskite solar cells by introducing Ag nanopartices and ITO AR layers
Source: Sci Rep. 2021 Jul 15;11:14550. doi: 10.1038/s41598-021-93914-1 (PMC8282636; doi:10.1038/s41598-021-93914-1)
Supplement: Supplementary file 1 — Supplementary Information. [file 41598_2021_93914_MOESM1_ESM.docx]

Improved optical properties of perovskite solar cells by introducing Ag nanopartices and ITO AR layers

**Yangxi Chen,^1^ ChaolingDu,^1,2,^*Lu Sun,^1^ Tianyi Fu,^1^ Ruxin Zhang,^1^ Wangxu Rong,^1^ Shuiyan Cao,^1,2^ Xiang Li,^1^Honglie Shen,^2,3^& Daning Shi^1,2^**

*^1^College of Science, Nanjing University of Aeronautics and Astronautics, Nanjing 211100, PR China*

*^2^Key Laboratory of Aerospace Information Materials and Physics, Ministry of Industry and Information Technology, Nanjing 210016, China*

*^3^College of Materials Science and Technology, Nanjing University of Aeronautics and Astronautics, Nanjing 211100, PR China*

**[cldu@nuaa.edu.cn](mailto:cldu@nuaa.edu.cn)*

**Supplementary Figures and Text**

**
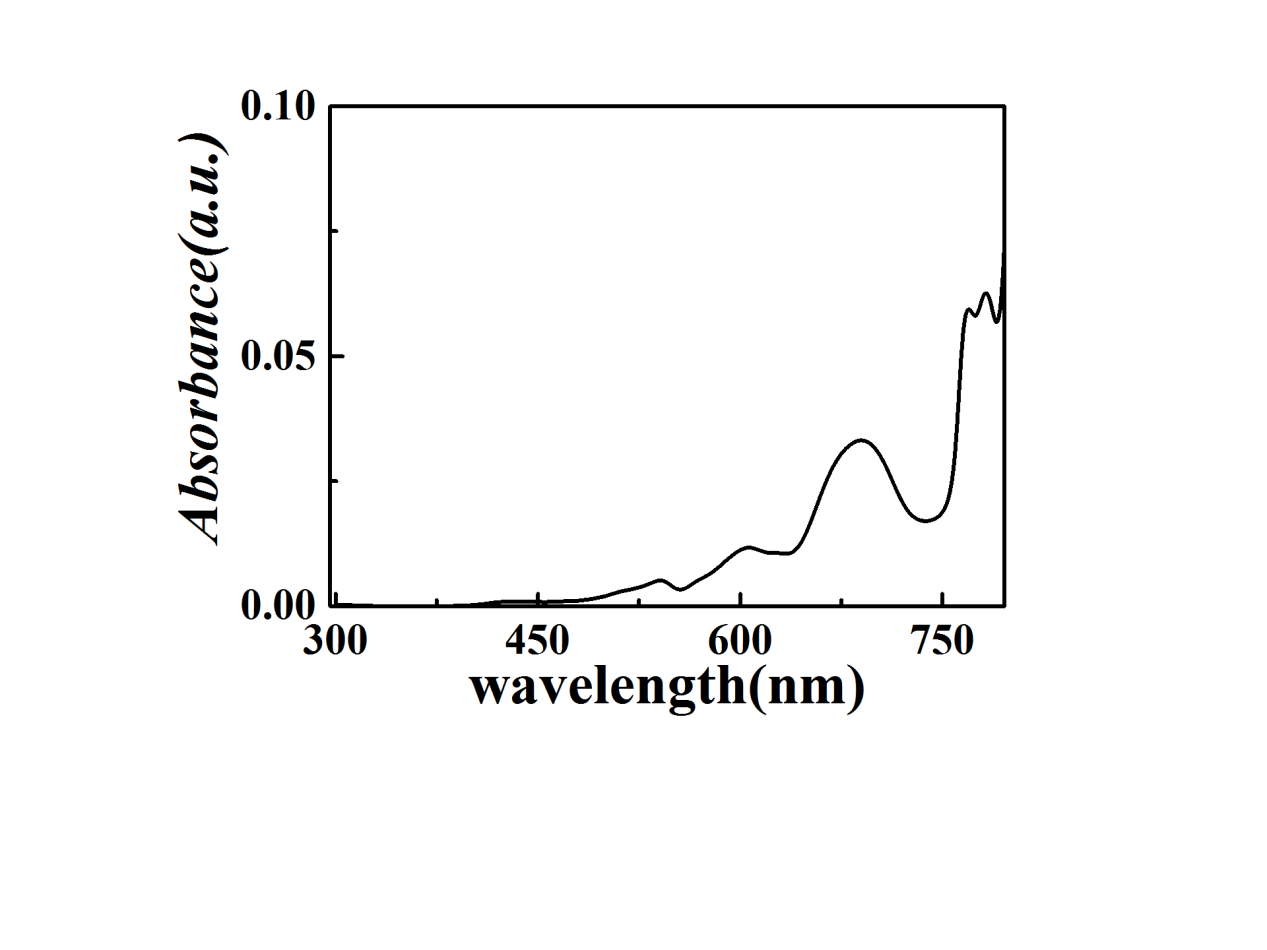
**

**Fig. S1** The absorption spectra of the concerned Ag nanocube

in the optimized solar cell of Case V.

**
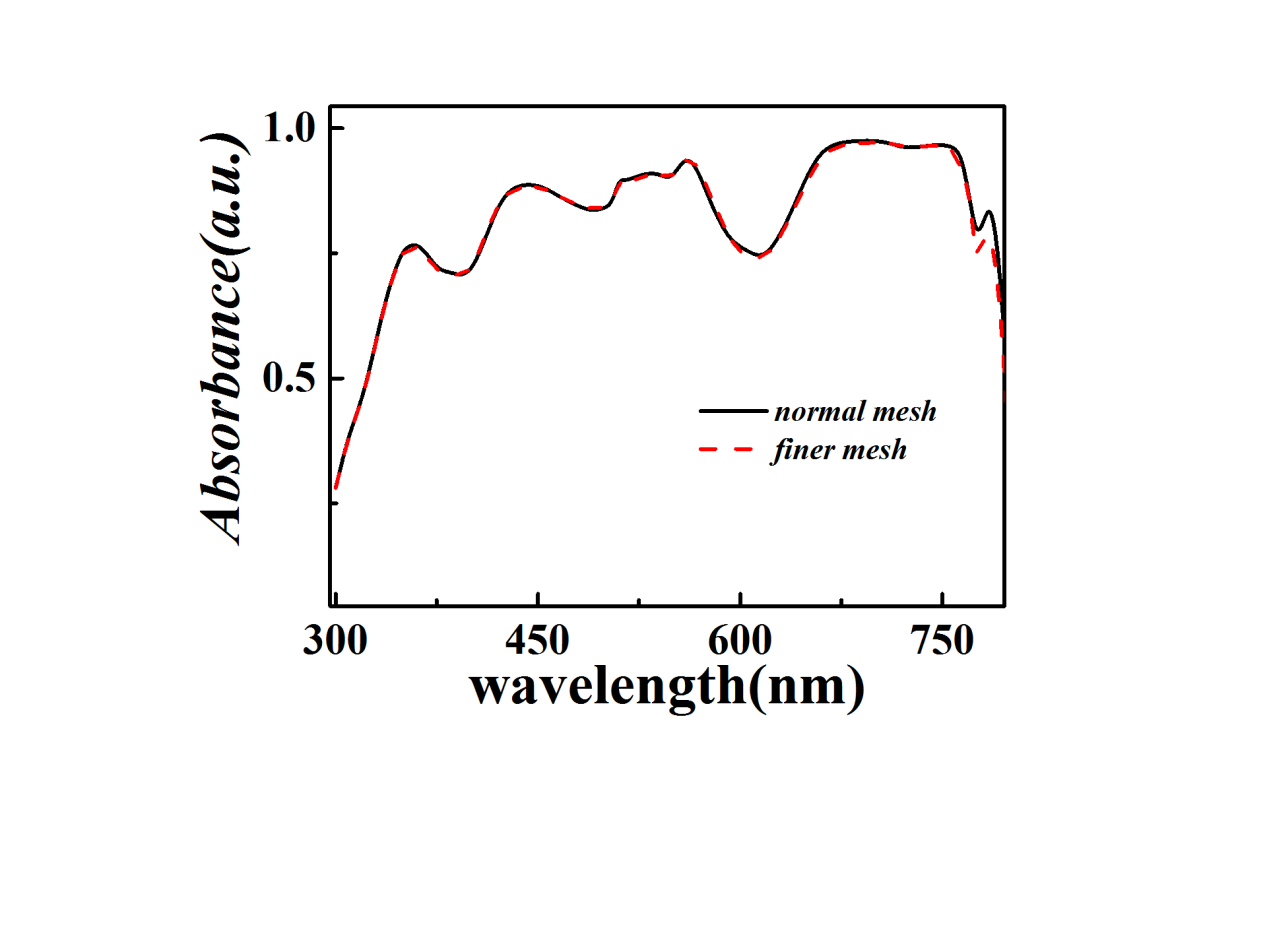
Fig.S2** The absorption spectra comparison between the normal and finer mesh.


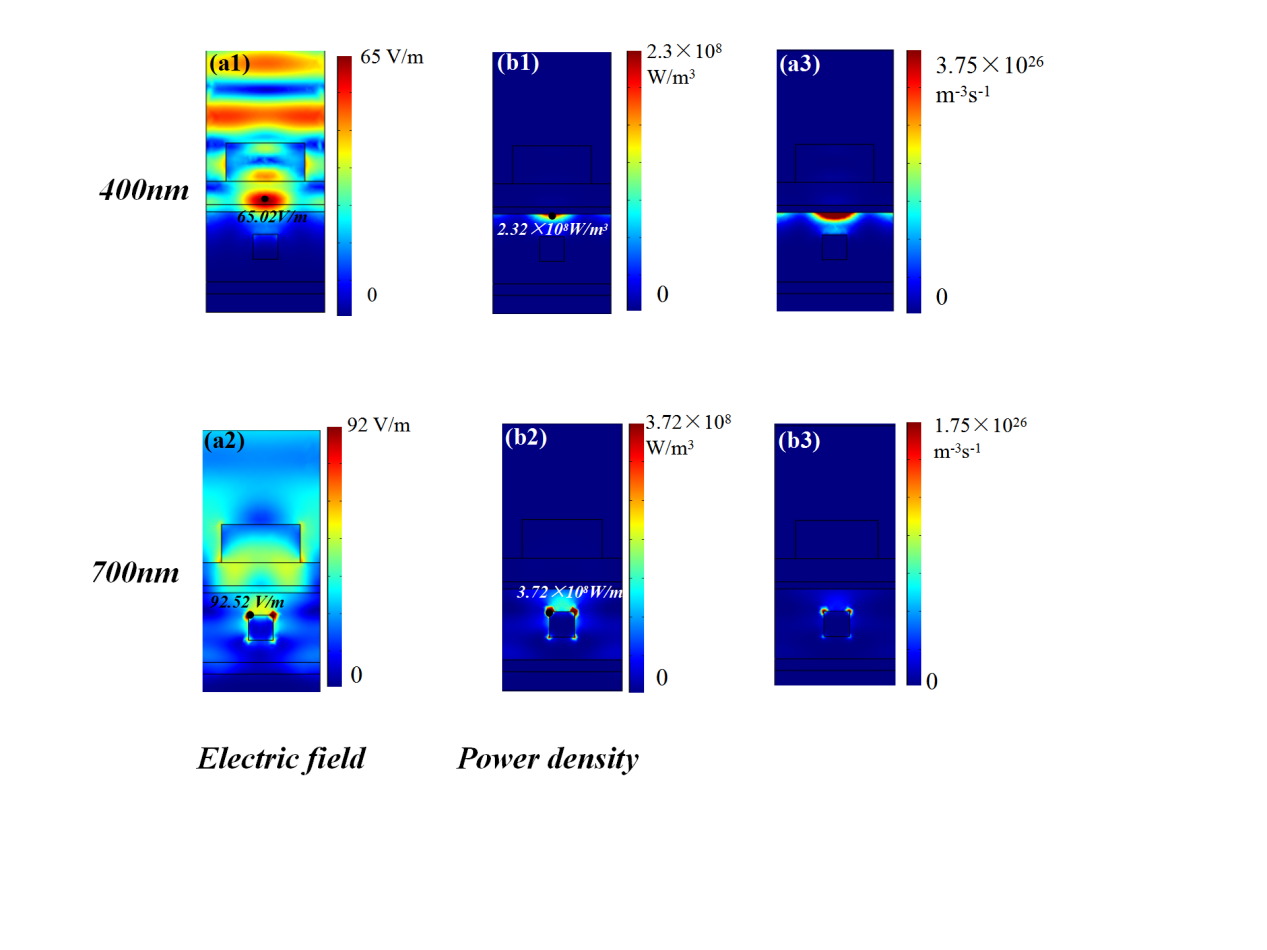


**Fig.S**3 The FEM simulated distribution maps of electric field (a1, b1) and power density (a2, b2) of optimized cell of Case V at 400nm and 700nm, respectively.


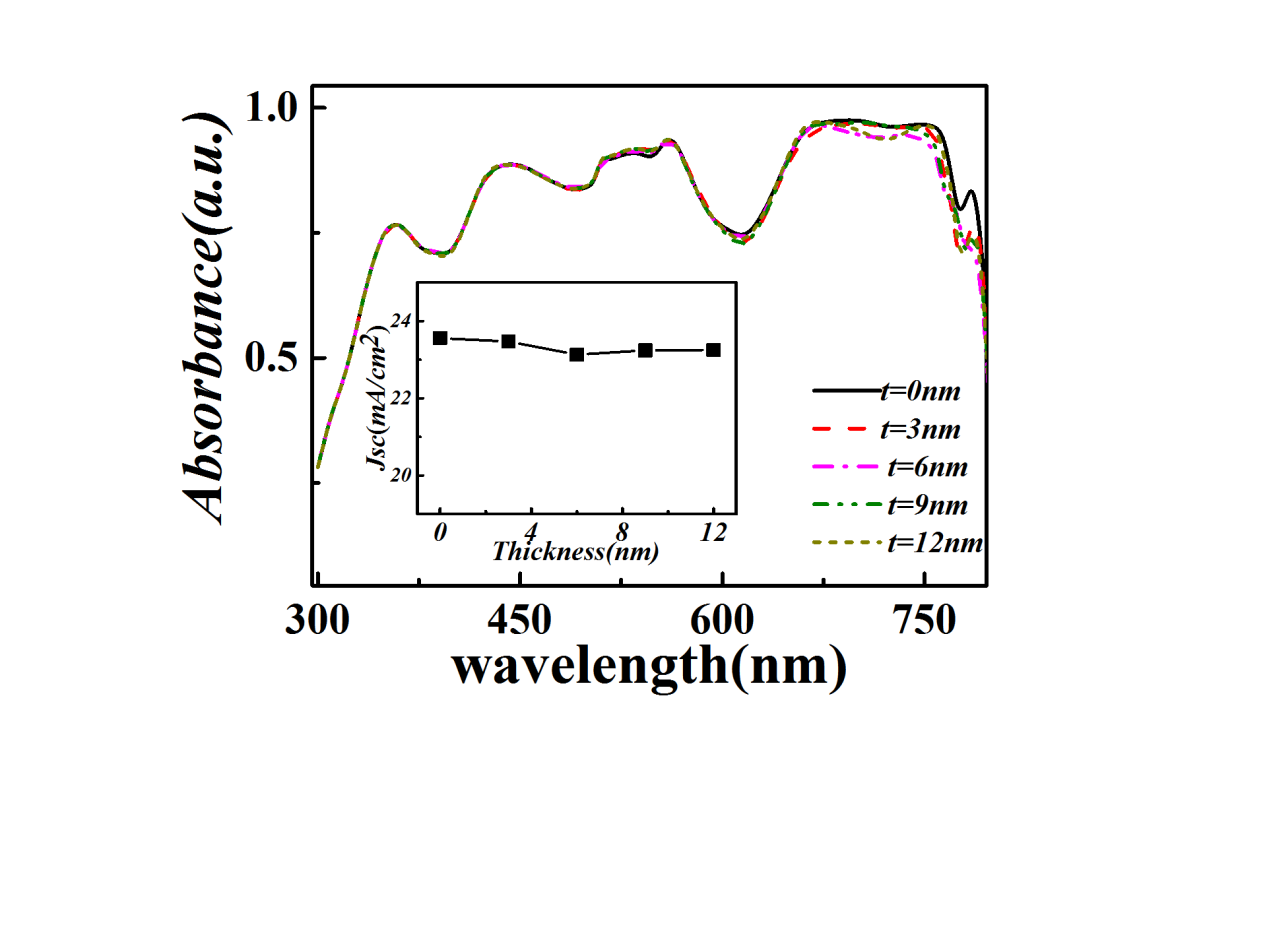


**Fig.S4** The absorption spectra of the optimized solar cell of Case V with which Ag nanocube is encapsulated with different thicknesses (*t*) of TiO_2_.


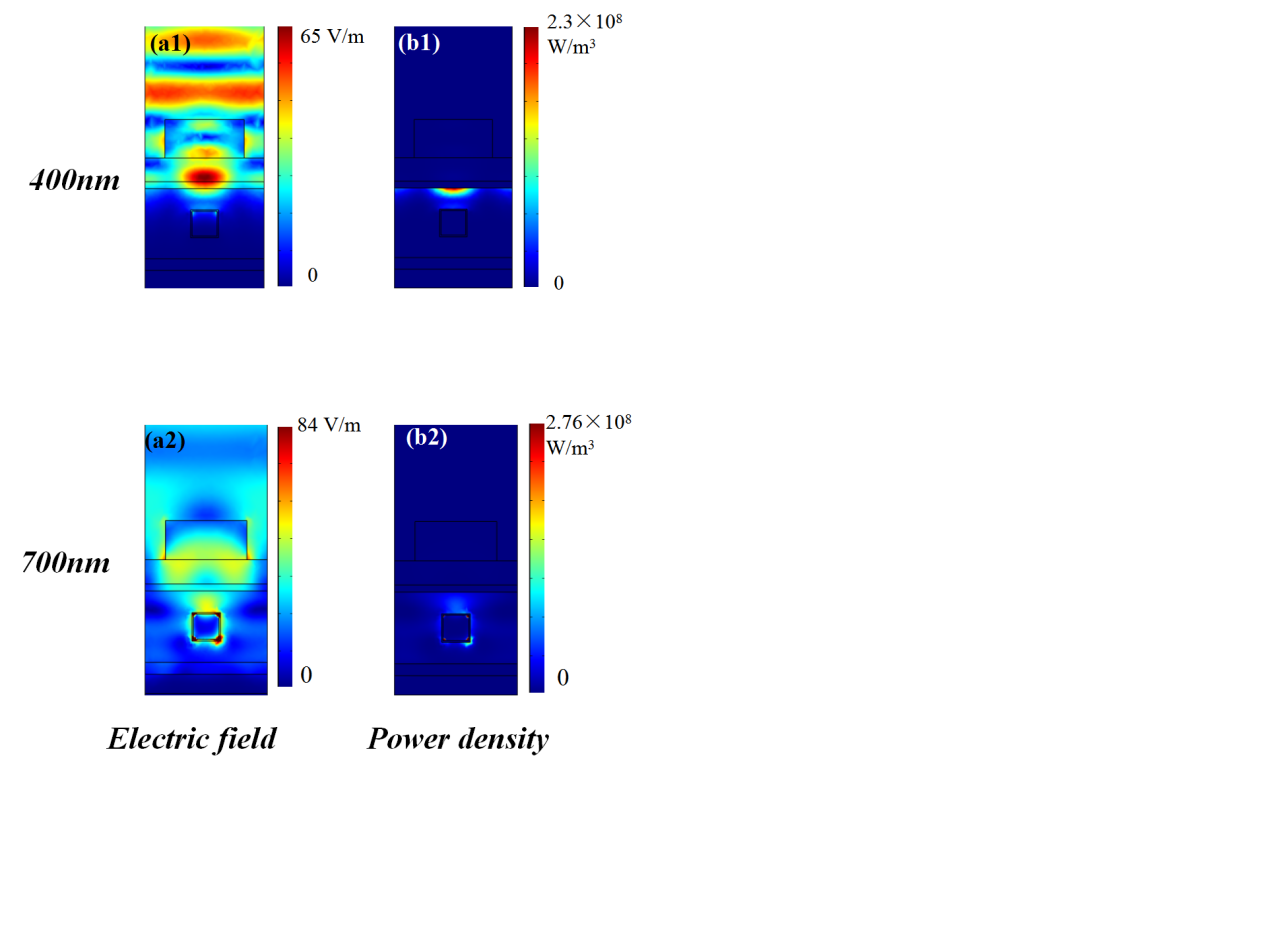


**Fig.S5** The FEM simulated distribution maps of electric field (a1, b1) and power density (a2, b2) at 400nm and 700nm for the optimized solar cell of Case V coated with 12nm TiO_2_ nanoshell.
